# Supplementary material for: Causal Relationship between Aging and Anorexia Nervosa: A White-Matter-Microstructure-Mediated Mendelian Randomization Analysis
Source: Biomedicines. 2024 Aug 16;12(8):1874. doi: 10.3390/biomedicines12081874 (PMC11351342; doi:10.3390/biomedicines12081874)
Supplement: Supplementary file 1 [file biomedicines-12-01874-s001.zip › Supplementary Figure/Figure S8/Leaveoneout plot of MR analysis for TL to ubm-b-1510.pdf]

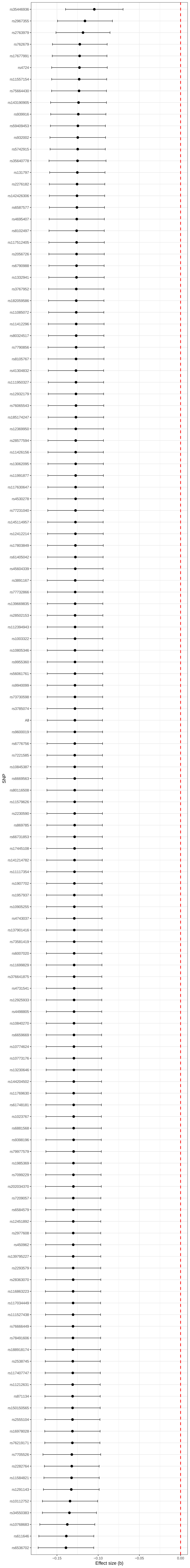

SNP
Effect size (b)
-0.15
-0.10
-0.05
0.00
rs35446936
rs2967355
rs2763979
rs762679
rs17677991
rs4724
rs11557154
rs75664430
rs143190905
rs939916
rs59409453
rs932002
rs5742915
rs35640778
rs131797
rs2276182
rs142426306
rs6587577
rs4695407
rs8102497
rs117512405
rs2056726
rs6790988
rs1332941
rs3767952
rs182059586
rs11085072
rs11412296
rs80324517
rs7790856
rs8105767
rs41304832
rs111950327
rs12932179
rs76065543
rs185174247
rs12369950
rs28577594
rs11426156
rs13062095
rs11991877
rs117630647
rs4530278
rs77231040
rs145114957
rs12412214
rs17803849
rs61405042
rs45604339
rs3891167
rs77732866
rs139669835
rs28502153
rs112394943
rs1003322
rs10805346
rs9955360
rs56061761
rs9940099
rs73730598
rs3785074
A8
rs9600019
rs6776756
rs7221585
rs10845387
rs6669563
rs80116508
rs11579626
rs2230590
rs869785
rs66731853
rs17445108
rs141214782
rs11117354
rs1907702
rs1957937
rs10905255
rs4743037
rs137901416
rs73581419
rs6007020
rs11699829
rs376641875
rs4731541
rs12925933
rs4498805
rs10840270
rs6659669
rs10774624
rs10773176
rs13230646
rs144204502
rs11769630
rs61748181
rs1023767
rs6881568
rs9398196
rs79977579
rs1985369
rs7099229
rs202034370
rs7209057
rs6584579
rs12451892
rs2977608
rs450962
rs139795227
rs2293579
rs28363070
rs116863223
rs117034449
rs111527438
rs76666449
rs78491606
rs188918174
rs2538745
rs117407747
rs11212631
rs871134
rs150150565
rs2555104
rs16978028
rs76219171
rs7705526
rs2282764
rs11584821
rs1291143
rs10112752
rs34550383
rs10768683
rs611646
rs6536702
